# Estimating Physical Activity Energy Expenditure with the Kinect Sensor in an Exergaming Environment

**DOI:** 10.1371/journal.pone.0127113

**Published:** 2015-05-22

**Authors:** David Nathan, Du Q. Huynh, Jonas Rubenson, Michael Rosenberg

**Affiliations:** 1 School of Sport Science, Exercise and Health, The University of Western Australia, Perth, Australia; 2 School of Computing Science and Software Engineering, The University of Western Australia, Perth, Australia; 3 Biomechanics Laboratory, Department of Kinesiology, The Pennsylvania State University, University Park, U.S.A.; Purdue University, UNITED STATES

## Abstract

Active video games that require physical exertion during game play have been shown to confer health benefits. Typically, energy expended during game play is measured using devices attached to players, such as accelerometers, or portable gas analyzers. Since 2010, active video gaming technology incorporates marker-less motion capture devices to simulate human movement into game play. Using the Kinect Sensor and Microsoft SDK this research aimed to estimate the mechanical work performed by the human body and estimate subsequent metabolic energy using predictive algorithmic models. Nineteen University students participated in a repeated measures experiment performing four fundamental movements (arm swings, standing jumps, body-weight squats, and jumping jacks). Metabolic energy was captured using a Cortex Metamax 3B automated gas analysis system with mechanical movement captured by the combined motion data from two Kinect cameras. Estimations of the body segment properties, such as segment mass, length, centre of mass position, and radius of gyration, were calculated from the Zatsiorsky-Seluyanov's equations of de Leva, with adjustment made for posture cost. GPML toolbox implementation of the Gaussian Process Regression, a locally weighted k-Nearest Neighbour Regression, and a linear regression technique were evaluated for their performance on predicting the metabolic cost from new feature vectors. The experimental results show that Gaussian Process Regression outperformed the other two techniques by a small margin. This study demonstrated that physical activity energy expenditure during exercise, using the Kinect camera as a motion capture system, can be estimated from segmental mechanical work. Estimates for high-energy activities, such as standing jumps and jumping jacks, can be made accurately, but for low-energy activities, such as squatting, the posture of static poses should be considered as a contributing factor. When translated into the active video gaming environment, the results could be incorporated into game play to more accurately control the energy expenditure requirements.

## Introduction

Physical inactivity and a sedentary lifestyle are significant risk-factors for many diseases including cardiovascular disease, diabetes and cancer [1]. New and novel human computer interaction devices, such as active videogames, are recent developments in the interactive entertainment industry which have potential health benefits through the requirements to be physically active during screen-based games [2]. Compared to seated gaming, active gaming has been demonstrated to have a higher cardiovascular response and energy expenditure [3]. For children and adults who play active video games, a level of moderate intensity exercise has been shown to occur over a relatively wide range of gaming consoles and games [4, 5]. Greater physical activity intensity achieved during active video game play has been attributed in general to active video games that require whole body, compared with partial body movements [6]. Furthermore, movements that require a sustained higher intensity, such as boxing and dancing games, also result in higher levels of energy expenditure [7, 8]. However, to achieve an acute cardiovascular benefit from active video gaming, a high intensity bout of 15 minutes is required, and there appear very few games, if any, that achieve this level of intensity [9]. If active video games are to contribute to a range of health outcomes, including cardiovascular health, a more sophisticated understanding of how game players achieve physical activity intensity during game play is required.

Active video games typically comprise a gaming console, screen and a motion controller. Until recently, most gaming controllers were tethered to the console and/or player and required tactile feedback to play games [10]. These interfaces, commonly known as exertion interfaces, include hand held controllers (Wii remote), force platforms (Wii balance board), and included accelerometers that deliberately encourage physical effort from the user to achieve game outcomes.

Most measurements of physical activity energy expenditure during active video game play are currently made using heart rate monitors, accelerometers [11] and portable gas analysers attached to participants during game play. While appropriate to measure overall energy expenditure, none of these devices is capable of disintegrating total energy expenditure into the energy expended by individual body segment movements, movements that are used to move the body centre of mass, nor to differentiate between energy expended for movement and energy expended to maintain posture. However, advances in optical sensor technology, particularly at the consumer level through the Microsoft Xbox Kinect, provides new and novel ways of estimating total energy expenditure and its components during active video game play [12].

The Kinect sensor introduced in 2010 together with the release of its software development kit (SDK) makes it possible for software developers to acquire a skeletal model of the user in real-time with no calibration needed [13]. Unlike other optical marker-based motion analysis systems where the user must have markers attached at various body joints, the Kinect is a marker-less motion capture system. This is achieved through the implementation of a cost-effective depth camera using a technique called structured-light [14], and an object-recognition approach to the human pose estimation problem [13]. Because the capturing technique is non-intrusive, the user can move more freely and naturally when measurements are taken. Furthermore, marker-less motion capture has the ability to detect some of the movements that cannot be detected by accelerometers—for example, the upper body movements [7].

The kinematics measured by the Kinect can be used to estimate the mechanical work of the body during motion. The physiological energy expenditure (PE) of the skeletal muscles during motion can, in principle, be obtained from the mechanical energy of movement; however, a range of factors can affect this relationship, making accurate predictions of PE expenditure difficult [15, 16]. Methods of modelling the relationship between PE and mechanical work have been explored extensively (see the work of Ingen Schenau and Cavanagh [17]), but there is currently no accepted method to account for this relationship [18]. Nevertheless, there are advantages in using mechanical work measurements to estimate PE in active video gaming, since PE can be partitioned between that used to move the body centre of mass and that used to move the limbs relative to the centre of mass. Another benefit from using mechanical work is that it is a relatively simple and interpretable measurement from which features can be extracted. Thus, some of the limitations that come from using mechanical work for PE estimation may be reduced by using large samples of data along with algorithmic modelling techniques for predictive statistical models [19].

We propose to use marker-less motion capture to predict energy expenditure using predictive algorithmic models. Our aim is to estimate PE from the mechanical work of body segments using a multivariate model during exercise using marker-less motion capture. This is achieved using the following three steps:

1. Capturing mechanical work and metabolic cost for a range of exercises of varying intensity and movement type;
2. Deriving biomechanically appropriate features from mechanical work; and
3. Building a predictive multivariate model using nonparametric regression based on the derived features.

## Materials and Methods

### Subjects

Nineteen students (16 male; 3 female) from The University of Western Australia were recruited to participate in the study. The only exclusion criterion was students who were unable to perform moderate intensity exercise. Table 1 shows participants’ height, weight, age and BMI. The participants were requested to be barefooted with their weight measured using a standard scale calibrated to the nearest 0.05 kg and their stature measured using a stadiometer to the nearest 1 cm.

**Table 1:** Characteristics of the 19 subjects (16 male; 3 female).

Characteristic (n = 19	Median	Max.	Min.
Mass (kg)	74.10	92.20	52.00
Stature (cm)	177	186	161
Age (years)	23	33	19
BMI (kg/m^2^)	24.40	31.17	19.81

### Study Design

A repeated measures experiment was designed to model the relationship between metabolic energy consumption and mechanical work for a variety of movements that exhibited different forms of mechanical work. A total of four fundamental movements were tested in randomized order with each being repeated for a minimum of four minutes to reach steady-state exercise. These movements were arm swings, standing jumps, body-weight squats and jumping jacks, with the timing of each regulated by a metronome.

All participants were adults who provided written consent to participate in the study. Approval to conduct this research was granted by the Human Research Ethics Committee of The University of Western Australia (RA/4/1/6310).

### Experimental Procedure

Participants attended The University of Western Australia Exergaming Laboratory to complete the experiment. Upon arrival, participant’s height and weight were measured, and they were acclimatized to the testing environment.

**Fig 1:**
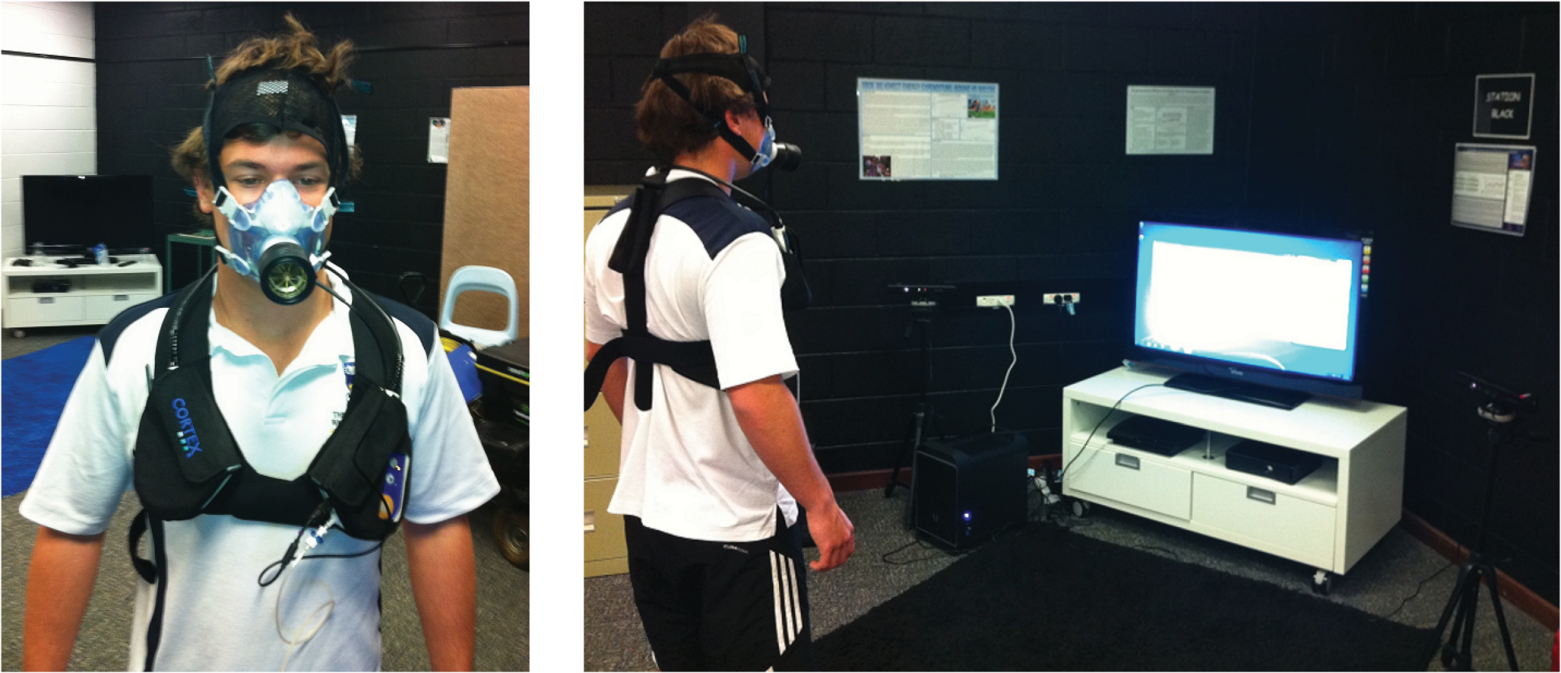
The Cortex Metamax 3B automated gas analysis system.

The rate of oxygen consumption and carbon dioxide production was measured using a Cortex Metamax 3B automated gas analysis system, which has previously been validated against the Douglas Bag Method [20–23]. The experimental setup with the use of the Cortex system is shown in Fig 1. The metabolic system was calibrated using a 5 litre volume syringe and a standardized gas mixture. Once fitted with a neoprene mask, each participant was required to stand still as the metabolic system was calibrated to the ambient temperature, pressure and gas concentration. The metabolic system returned breath-by-breath measurements of the rate of oxygen consumption ($\dot{\text{V}}{\text{O}}_{2}$) and the rate of carbon dioxide expiration ($\dot{\text{V}}{\text{CO}}_{2}$) in litres per minute.

After calibration, the participants were required to stand still while breathing normally for a further three minutes to record a standing resting metabolic rate (stRMR). We used a three-minute measurement period since our participants had been standing quietly for several minutes prior to recording and thus were regarded to be at physiological steady state. A three-minute duration was assessed to be sufficient for the recording equipment to measure metabolic rate accurately. It also allowed us to shorten slightly an already long experimental session for the participants.

To calculate the activity energy expenditure (EE), the stRMR was used as a baseline to be subtracted from the metabolic EE [15, 24]. A randomized order of the exercises was used for each participant. The timing of each exercise was regulated using a metronome with the speed being measured in beats per minute (BPM). Each exercise was repeated for a minimum of four minutes with a period of rest in-between that lasted until the participant’s $\dot{\text{V}}{\text{O}}_{2}$ returned to stRMR. The cadences were chosen based on a first approximation of the mechanical work required for each movement and the frequency of these movements that would result in an appropriate mechanical power and thus metabolic power ($\dot{\text{V}}{\text{O}}_{2}$). These initial approximations were made from an estimate of the translation of the mass of the body and limb segments and the approximate mass of the segments.

#### Standing Jumps at 120 BPM

Participants were required to vertically jump sub-maximally from standstill once every four beats (0.5 Hz) twenty times and rest for twenty beats. This resulted in five lots of twenty jumps with ten second breaks in-between over the four minutes. The jump frequency and intervals between jumps was made to ensure that the physical activity remained aerobic. This exercise was chosen to emphasize the mechanical work of the lower limbs to raise the centre of mass.

#### Arm Swings at 112 BPM

Participants were required to simultaneously swing both arms horizontally apart, return the arms to the side of the body, swing the arms forward, and then return the arms to side. The duration of each of these movements was one beat (0.53 s; 1.89 Hz). This exercise was chosen to emphasize the mechanical work of the upper limbs.

#### Squats at 72 BPM

Participants were required to lower their body by bending their knees and holding their position for eight beats (6.67 s) and then stand upright for another eight beats (6.67 s). This exercise was chosen to test to what extent the energy expenditure of isometric muscle contractions to counter gravity would affect our predictive model.

#### Jumping Jacks at 76 BPM

Participants were required to jump and move their feet apart while raising their arms above their head on the first beat, and jump and bring their feet together and lower their arms to side on the next beat (0.63 Hz). This exercise was chosen to have the maximum amount of mechanical work to raise the centre of mass and across all limb segments.

A 15-second moving average was used to smooth the $\dot{\text{V}}{\text{O}}_{2}$ measurements. The value of 20.964 (kJ) was used as the energy equivalence of one litre of consumed oxygen [25] and was used to compute a metabolic power (kW). The average of the three minutes of standing was used as the baseline subtraction and the average of the last minute, not including the final data point, was used as the steady-state energy expenditure of the exercise. Metabolic work (kJ) was computed by integrating the metabolic power trace across this last minute of exercise.

### Motion Data Collection

#### Structure of the motion data

**Fig 2:**
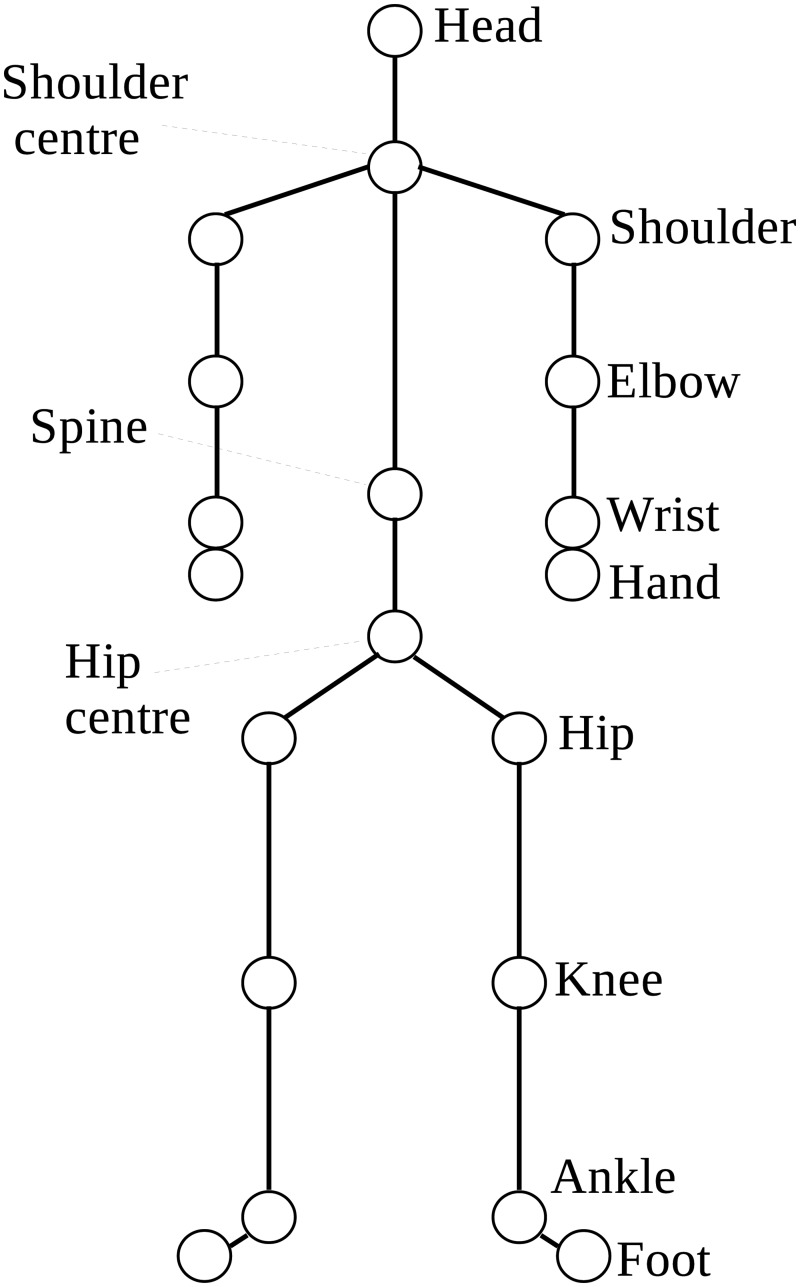
The 20 body joints of the human skeletal model.

The existing Microsoft Kinect for Windows SDK provides an API and access to the data streams of the camera which produces 30 frames of data per second. The skeleton stream tracks 20 joints by default (Fig 2). The position of each joint is stored as three floating point numbers that represent the XYZ coordinates in metres from the camera.

The data structure of the human skeletal model is a hierarchical tree, where the root of the tree is at the centre of the two hip joints. This joint is referred to as the *root joint*. A body joint *J*_1_ is said to be the *parent joint* of a body joint *J*_2_ if both joints are directly connected in the skeletal model and if *J*_1_ is closer to the root joint than is *J*_2_. Alternatively, *J*_2_ is said to be a *child joint* of *J*_1_. A bone segment is therefore specified by two adjacent joints in the hierarchy, with the tracked information stored in the child joint. The orientation of each bone segment is the transformation in space from its parent’s bone coordinate frame. Thus, there are 19 relative orientations in the kinematic skeleton returned by the Microsoft API (application programming interface) from the SDK. In addition, the absolute orientation and displacement of the player with respect to the camera stored in the root joint are also returned. These parameters are returned as a 4 × 4 rigid transformation matrix. The SDK uses single-precision floating point numbers and a pose recognition algorithm [13] to determine the human pose. Problems such as occlusion and infrared interference may affect the accuracy of the skeletal model parameter values.

The body was modelled as a 14 segment rigid body, with 3 segments for each limb, as well as a trunk and head segment. An important difference between the pose recognition algorithm of Shotton et al. [13] and marker-based motion capture systems is that their algorithm does not enforce individually calibrated constraints on the kinematic skeleton. Thus, properties such as bone segment length can vary between different poses of the same individual. To overcome this limitation we use the adjustment procedure on the lengths of body segments [26] to define the endpoint of a segment rather than using the *child joint* position directly.

#### Calibration for the Kinect Cameras

Based on the Windows API, we developed a software system that combines the motion data from two Kinect cameras. Our software system involves two Kinect cameras placed at 60 degrees apart in front of the participant so as to minimize self-occlusion and interference. We found that the interference between the Kinect cameras is minimized for such a setup as they observe slightly different body parts of the participant. The motion data stored in the data file is in binary format containing the twenty joint positions in XYZ coordinates, a validity character for each joint, twenty bone orientations in YXZ Euler angles and a timestamp for each frame.

To combine the data from the two cameras, the participant is required to stand still with their arms held apart so that they make a straight horizontal line. This assumes that both cameras are at the same height so they differ only by a translation and a rotation about the vertical axis. The distance between the left wrist and right wrist is measured for each Kinect camera. To ensure that the wrist positions estimated by both cameras are accurate for calibration, the software system checks and imposes that the difference between the two distance values is less than 10cm. These wrist positions are then used to determine the relative angle and displacement of the second camera with respect to the first. This allows the joint positions from the second camera to be transformed into the coordinate system of the first and vice versa. If the joint is being tracked by camera 1 but not camera 2 then the value for camera 1 is used and vice versa. If the joint position is valid in both cameras then the average of the two is taken; if it is invalid in both then camera 1’s position is used. These different situations are represented in the validity character in the data file. For the orientations of the bone segments, the observations from camera 1 were always used.

### Segmental Mechanical Work Modelling

#### Overview

Forces generated by individual muscles are generally unknown and are difficult to directly observe in human movement [27]. What is observed is the motion that occurs from the net force acting on the body, and so while the mechanical work done by the individual muscle forces cannot be calculated, the mechanical work done on the body can be calculated from changes in its mechanical energy. Our approach involved using different parts of the mechanical work as separate variables in a multivariate model to predict metabolic energy cost.

#### Our Model

We adapt the approach of Willems et al. [28] that includes both external work (the work required to move the body centre of mass, as measured from kinematics) and internal work (the work required to move the body segments relative to the centre of mass). We also adapt their method to model the transfer of mechanical energy between adjacent segments. While Willems et al. [28] only calculated segment energy transfer for the lower limbs, we extend these calculations to the upper limbs. Since muscles expend energy when they lengthen as well as when they shorten, we also separate mechanical work into positive and negative components. This is important since muscles are considered less efficient for positive work (*concentric contractions*) than negative work (*eccentric contractions*) [29]. By separating positive and negative work into different variables, the predictive model can learn the relative efficiencies solely from the sample data. Estimations of the body segment properties, such as segment mass, length, centre of mass position, radius of gyration, were calculated from the adjusted *Zatsiorsky-Seluyanov’s equations* of de Leva [26].

#### External work

The energy at any instant of time *t* associated with the external work of the centre of mass of the whole body (COM_wb_) is defined as

$$\begin{array}{c} E_{\text{COM}}\left(t\right)=MgH\left(t\right)+\frac{1}{2}MV_{\text{cg}}{\left(t\right)}^{2}\;, \end{array}$$ (1)

where *M* is the total mass of the body, *g* is the gravitational acceleration, *H* is the height of COM_wb_ and *V*_cg_ is the linear velocity of the COM_wb_ relative to the environment.

From the centre of mass position computed from Zatsiorsky-Seluyanov’s equations and the coordinates of the root joint of skeletal model of the participant, an offset position along the spine of the skeletal model can be obtained. At any time instant, we approximate the COM_wb_ of the participant to be the coordinates of the root joint plus this offset. The linear velocity *V*_cg_ is approximated from the finite differences of COM_wb_ over consecutive time instants. The calculations assess the work of the limb relative to the COM and thus take any changes in the movement of the COM into account. In our software system, variable *H* corresponds to the vertical component of COM_wb_ above the floor plane.

Let *W*_ext_(*t*) = *E*_COM_(*t* + 1) − *E*_COM_(*t*). Then

- *positive external work* is defined to be the sum of positive *W*_ext_(*t*) values over the exercise recording period;
- *negative external work* is defined to be the sum of negative *W*_ext_(*t*) values over the exercise recording period.

#### Internal work and energy transfer between limb segments

The energy at any instant of time *t* associated with the internal work of a segment *i* is defined as

$$\begin{array}{c} E_{\text{int},i}\left(t\right)=\frac{1}{2}m_{i}V_{\text{r},i}{\left(t\right)}^{2}+\frac{1}{2}m_{i}K_{i}^{2}\omega_{i}{\left(t\right)}^{2}\;, \end{array}$$ (2)

where *m*_*i*_ and *ω*_*i*_ are, respectively, the mass and angular velocity of the *i* ^th^ segment, *V*_r, *i*_ is the relative velocity of the centre of mass of the *i* ^th^ segment (i.e., COM_*i*_) to COM_wb_, and *K*_*i*_ is the radius of gyration from COM_*i*_.

From the 3D coordinates of the two end points of the *i* ^th^ body segment, the COM_*i*_ is taken to be the centroid of these end points. For instance, the coordinates of the shoulder and the elbow joints are used to compute the coordinates of the upper arm segment. Variable *m*_*i*_ described above is computed from Zatsiorsky-Seluyanov’s equations. Similar to the linear velocity *V*_cg_, the linear velocity of segment COM_*i*_ can be computed in a similar manner. Taking into account the movement of the whole body, the relative velocity *V*_r, *i*_ can be estimated. The angular velocity *ω*_*i*_ of the *i* ^th^ is computed in a similar manner.

Having obtained all the necessary terms, the internal work *E*_int, *i*_ for each *i* ^th^ segment can be computed for each time instant using Eq (2). The equation assumes a complete transfer of energy between translational and rotational kinetic energy within a segment. Energy transfer is not permitted between the trunk and adjacent limb segments; however, energy transfer is permitted between adjacent segments of the same limb. For example, if the thigh has negative internal work and the shank and foot have positive internal work, then the thigh can only transfer energy to the shank.

Let *W*_int, *t*_(*t*) = *E*_int, *i*_(*t* + 1) − *E*_int, *i*_(*t*) be the internal work value for the *i* ^th^ segment of a specific limb. Then, after the internal energy transfer has been considered, the *positive* (or *negative*) *internal work* for that limb is defined to be the sum of *positive* (or *negative*) *internal work* values of all the limb’s segments over the exercise recording period. For the *positive* (or *negative*) *lower limb work*, this is simply the sum of the left and right lower limb positive (or negative) internal work. The positive and negative upper limb works are defined in a similar fashion.

**Fig 3:**
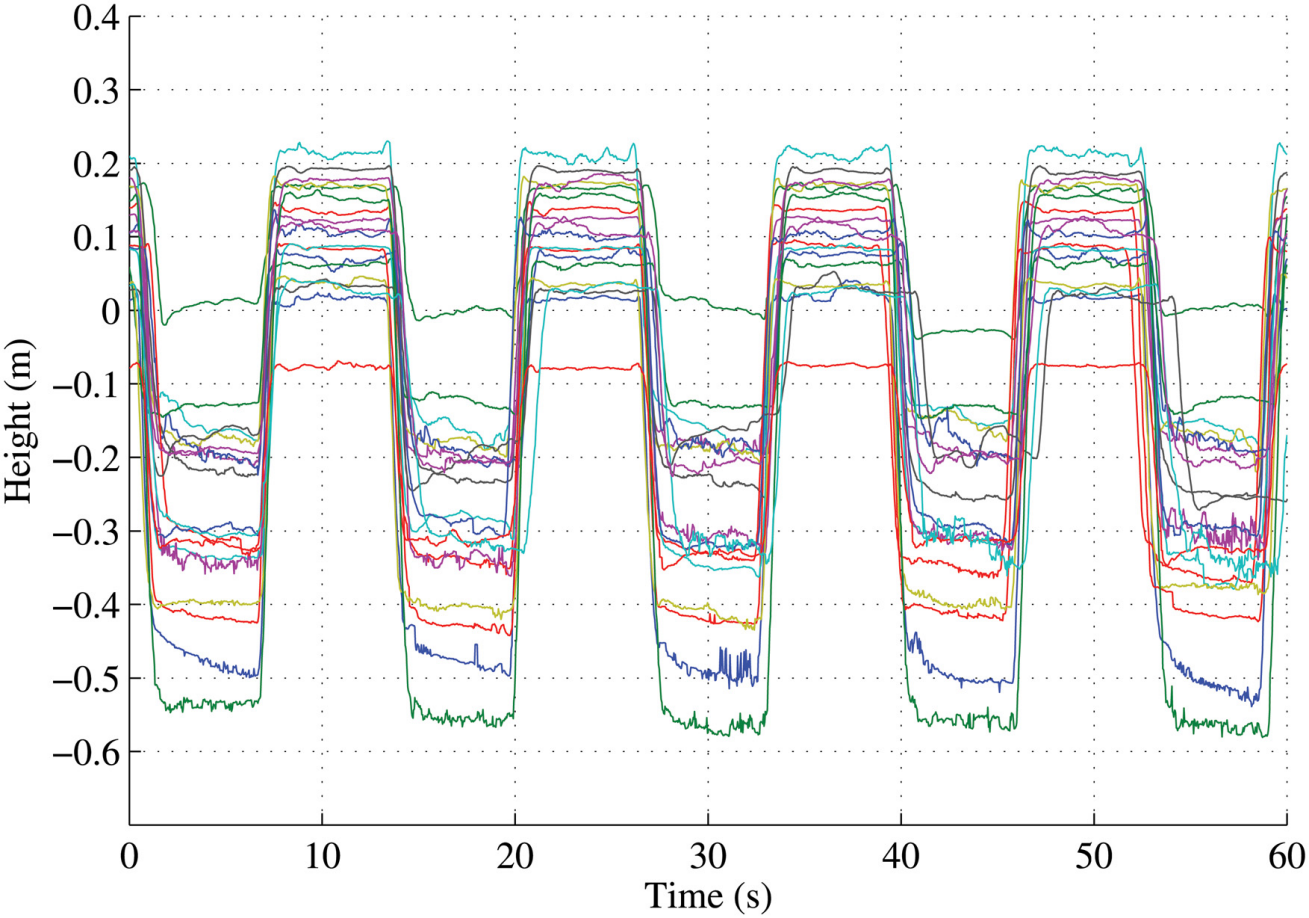
Temporal alignment of the hip centre joints of the participants for the squatting activity.

Putting all of these together we thus have six variables: positive external work, negative external work, positive lower limb internal work, negative lower limb internal work, positive upper limb internal work, and negative upper limb internal work. The last minute section of each recording was temporally aligned to ensure that the same cycle of the exercise was used across all participants for the mechanical work calculations. For example, the last minute of the squat exercise was aligned (Fig 3) so that the mechanical work of only 4.5 squat cycles was used. These 6 variables form the 6-dimensional (or 6D) feature vector for each data observation.

An added processing technique to the mechanical work features, namely, an estimation of *posture cost*, was also implemented. For the squat exercise in particular, a large component of the muscular energy expenditure would be due to the increase in joint torque at the knees and hips to maintain posture, yet no mechanical work would be observed. To overcome this, instead of mechanical work, we used a *posture cost* when the participant was stationary, i.e., when the velocity of COM_wb_ was smaller than a threshold for more than a specified number of frames. We used a velocity threshold of 0.05 m/s for a duration of 30 frames (i.e., 1 second). We define the *posture cost* to be the difference between the *default standing cost* of 1.5 watts per kilogram (a value commensurate with our observed standing costs and those of others [30]) and a *scaled standing cost*. The scaled standing cost was calculated as the default standing cost multiplied by the ratio of the sum of the left and right leg lengths to the sum of the left and right ankle-to-hip distances over the duration of the stationary period. Thus, the lower was the person’s centre of mass over a longer stationary period, the higher was the posture cost. This posture cost and the previous 6 variables together form a 7-dimensional (or 7D) feature vector for each data observation.

In addition to the 6D and 7D feature vectors above, we also computed the sum of the 6 variables above to yield a work value composed of all external plus internal work. This gave us a 1-dimensional (or 1D) feature vector for each data observation. This is similar to the approaches used to calculate total mechanical work [18, 31, 32]. We therefore have 3 types of feature vectors to represent the mechanical work of our data observations. With 19 subjects (see Table 1) participating in the experiments and each subject performed the 4 fundamental movements, there were 76 samples of (1D, 6D, and 7D) feature vectors and metabolic energy costs in our data collection.

### Predictive Models

To model some of the complexities involved in calculating metabolic cost from mechanical work, we implemented and compared three regression technique: Gaussian Process Regression (GPR) [33–35], a locally-weighted k-Nearest Neighbour Regression (KNNR) [36], and linear regression (LINR). We used the GPML toolbox implementation [37] of the GPR and the knnsearch function of Matlab for the KNNR.

#### Regression in machine learning

Given a training dataset of *n* observations {**x**_*i*_∣*i* = 1, ⋯, *n*} and corresponding target values {*y*_*i*_∣*i* = 1, ⋯, *n*}, the objective of regression in machine learning is to predict the target value y_*_ for a new input feature vector x_*_ Between the observations and the target values there is an unknown relationship, i.e., *f*(**x**) = *y*. The function *f*, which maps an observation **x** to a target value *y*, may be described parametrically if we have sufficient knowledge about the problem domain and the data; otherwise, a non-parametric approach would have to be sought where the form of function *f* is never explicitly modelled. For more in-depth discussion on regression in machine learning, we refer the reader to Bishop [38] and Prince [39].

#### Gaussian process regression

Gaussian process regression [33–35] is a nonparametric regression technique. In GPR, a Gaussian process (GP) places a prior over the space of functions for *f* without explicitly parameterizing *f*. A GP is completely specified by a mean function and a covariance function, cov(*f*(**x**), *f*(**x**′)), which by definition is: $\text{cov}(f(\text{x}),f({\text{x}}^{\prime }))=(f(\text{x})-\mu_{f}){\left(f({\text{x}}^{\prime })-\mu_{f}^{\prime }\right)}^{⊤}$ where *μ*_*f*_ is the mean of *f*(⋅). Our aim is to find a suitable *k*(**x**,**x**′) such that *k*(**x**,**x**′) = cov(*f*(**x**), *f*(**x**′)). The free parameters involved in the covariance function are known as *hyperparameters* [40].

In our case, we investigated the cases where each feature vector in {**x**_*i*_∣*i* = 1, ⋯, *n*} is a 1D, 6D, or 7D feature vector and each target in {*y*_*i*_∣*i* = 1, ⋯, *n*} is a metabolic energy cost. The value of *n* was 76. In our experiments, the feature vectors were normalized so that the mean and standard deviation for each dimension were 0 and 1, respectively. The same normalization was applied to the metabolic energy. For the covariance function, we found that the *dot product covariance function* was the most suitable:

$$\begin{array}{c} k\left(x,x^{\prime }\right)=\sigma_{0}^{2}+x\cdot x^{\prime }\;. \end{array}$$ (3)

The only hyperparameter here is $\sigma_{0}^{2}$, which represents a noise term in the samples.

Given that we are predicting the metabolic energy from noisy samples, we model *y* = *f*(**x**) + *ϵ*, where *ϵ* represents a Gaussian noise term (of 0 mean) in the metabolic cost estimation. Thus, putting all the target metabolic energy values into a vector, **y** = [*y*_1_, *y*_2_, ⋯, *y*_*n*_]^⊤^, we have $\text{cov}(\text{y})=K(X,X)+\sigma_{0}^{2}I$, where *X* denotes the collection of all the feature vectors **x**’s and *K*(*X*, *X*) is an *n* × *n* matrix storing the *k*(**x**_*i*_,**x**_*j*_) values. The metabolic energy for a new observed feature vector can then be predicted using the computed cov(**y**) matrix and *K*(x_*_, *X*) vector.

#### k-Nearest Neighbour

The k-nearest neighbour algorithm is a simple pattern recognition algorithm that finds the *k* nearest feature vectors in the training set to a given test vector, where *k* is a pre-defined positive integer. The locally-weighted regression simply takes the *k* targets that correspond to these *k* nearest vectors from the test vector x_*_ in the training set and weights them according to the inverse of their distances from the test vector. The weighted average of these targets gives a predicted target value y_*_. Mathematically, if *N*(x_*_) is the set of the *k* nearest neighbours of x_*_ and $\tilde{y}$ is the metabolic energy corresponding to the neighbour $\tilde{\text{x}}$ of x_*_, then

$$\begin{array}{c} y_{*}=\frac{\sum_{\tilde{x}\in N\left(x_{*}\right)}\tilde{y}\;w\left(\tilde{x},x_{*}\right)}{\sum_{\tilde{x}\in N\left(x_{*}\right)}w\left(\tilde{x},x_{*}\right)}\;, \end{array}$$ (4)

where $w(\tilde{\text{x}},{\text{x}}_{*})=1/d(\tilde{\text{x}},{\text{x}}_{*})$ is the weight for the neighbour $\tilde{\text{x}}$ of x_*_ and *d*(⋅, ⋅) can be any distance function. Other weighting functions that we tested include $w(\tilde{\text{x}},{\text{x}}_{*})=1/d^{2}(\tilde{\text{x}},{\text{x}}_{*})$ and $w(\tilde{\text{x}},{\text{x}}_{*})=\exp(-d^{2}(\tilde{\text{x}},{\text{x}}_{*})/b)$, where the value of *b* should be appropriately determined to ensure that all the weight values have reasonable magnitudes.

For the k-nearest neighbour regression (KNNR), the only free parameter is the value of *k*, which determines the cardinality of the set *N*(x_*_). KNNR does not need to have a training stage at all if the value of *k* is suitably determined by the user for the application. In the literature, various heuristic techniques (e.g., hyperparameter optimization [35]; minimization of the reconstruction error via cross validation) have been suggested to estimate an optimal value for *k* in the training stage.

#### Linear regression

Similar to KNNR where no prior training is required, the linear regression (LINR) algorithm produces the vector **α** = (*α*_1_, ⋯, *α*_*D*_), where *D* denotes the dimensions (1, 6, or 7) of the feature vectors, such that the total regression error is minimized:

$$\begin{array}{c} \sum_{i=1}^{n}{\left(\alpha^{⊤}x_{i}-y_{i}\right)}^{2}\;. \end{array}$$ (5)

In the training stage, the vector **α** is obtained from the training dataset. In the prediction stage, given a feature vector x_*_, the predicted y_*_ is computed as y_*_ = **α** ^⊤^ x_*_.

#### Cross validation

*Cross validation* is a technique for assessing the accuracy of an algorithm when applied to new unknown data. It provides a measure of the generalizability of a statistical model. To obtain statistics (e.g., the average regression error) on the performance of the algorithm, the dataset is parsed a sufficient number of times. In each parse, the dataset is partitioned into a *training set* and a *validation set* (or *test set*). The regression algorithm is trained on the training set and tested on the test set. The regression error on the test set can be computed since the ground truth of the target values *y*’s are known.

To assess the performance of the GPR, KNNR, and LINR algorithms, we used the *leave-one-out cross validation* (LOOCV) technique. From the *n* samples of **x**_*i*_ and *y*_*i*_, for *i* = 1, ⋯, *n*, that we collected, we took each (**x**_*i*_, *y*_*i*_) in turn to be the test set and the remaining samples to be the training set. So the training set in each parse was formed by leaving one sample out. This allowed us to obtain n regression errors from which we computed the *root mean squared error* (RMSE).

#### Concordance Correlation Coefficient and Paired t-test

The *concordance correlation coefficient* proposed by Lin [41] was designed for evaluation of reproducibility, i.e., how well two sets of scalar measurements computed from the same data differ. The concordance correlation coefficient (CCC) focuses on how the two sets of measurements deviate from the 45° line passing through the origin. The concordance correlation coefficient *ρ* is given by

$$\begin{array}{c} \rho =\frac{2\sigma_{12}}{\sigma_{1}^{2}+\sigma_{2}^{2}+\left(\mu_{1}-\mu_{2}\right)}\;, \end{array}$$ (6)

where *μ*_1_, *μ*_2_, $\sigma_{1}^{2}$, and $\sigma_{2}^{2}$ are the means and variances of the two sets of measurements, and *σ*_12_ is their cross correlation. We use the CCC to assess how the predicted metabolic energy values deviate from the ground truth metabolic energy values in the cross validation process. We then use a paired t-test via the Matlab ttest function to test whether there is a statistically significant difference in the predictions between methods. We set the *α*-value of the paired t-test to 0.05.

## Experimental results

### Metabolic results

**Fig 4:**
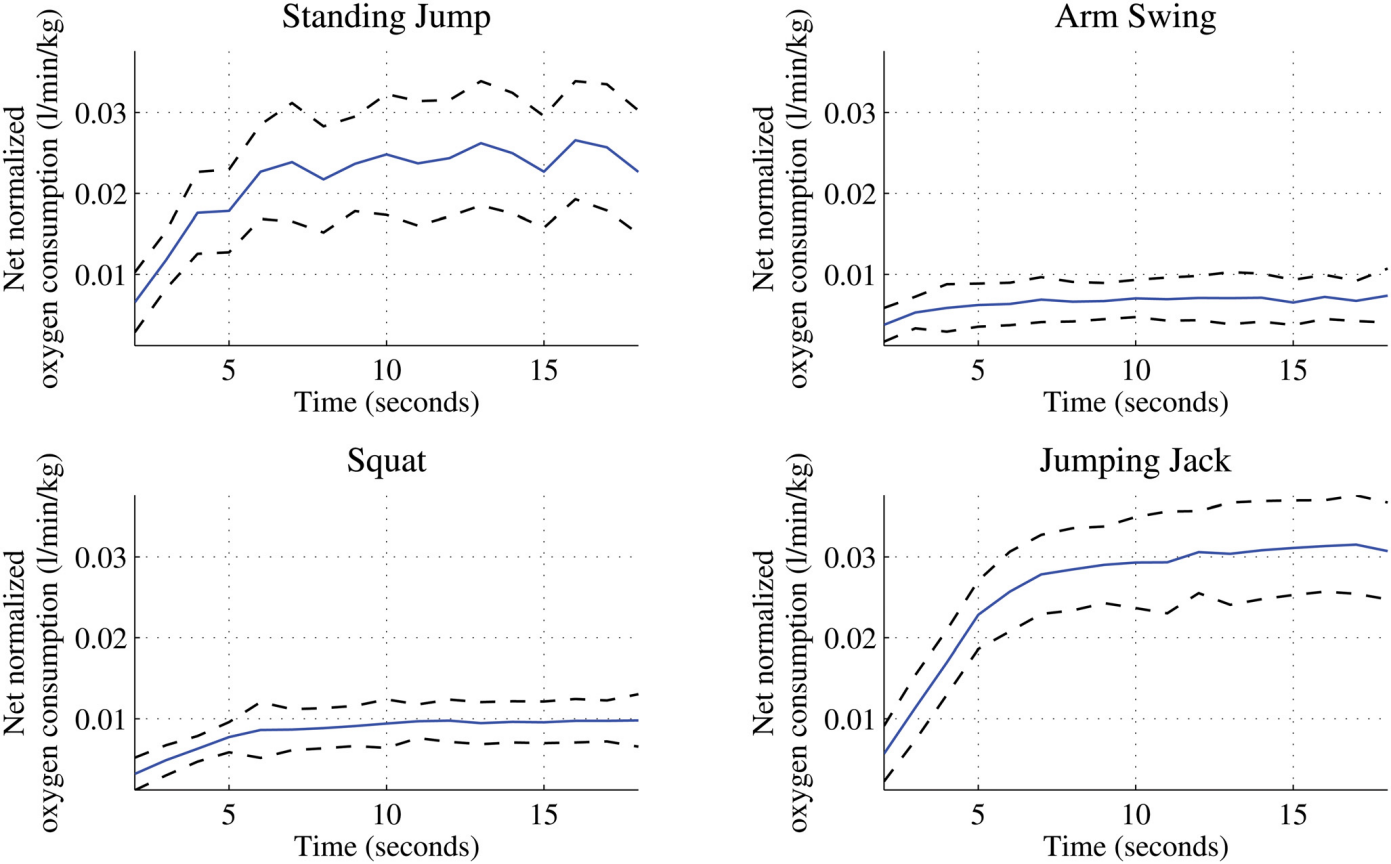
The average oxygen consumption of the four fundamental movements.

The mean net normalized oxygen consumption for the 19 participants is presented in Fig 4 for each activity by the blue curve with the one standard deviation above and below represented by the black dotted curve. The net normalized oxygen consumption, which is the difference between the gross oxygen consumption and the resting $\dot{\text{V}}{\text{O}}_{2}$ is the amount of $\dot{\text{V}}{\text{O}}_{2}$ attributed to the activity. It is evident from the figure that there is a distinct $\dot{\text{V}}{\text{O}}_{2}$ profile for each activity demonstrating that different intensity levels are represented.

Physiological energy expenditure was calculated from the energy equivalence of the average $\dot{\text{V}}{\text{O}}_{2}$ of the last minute of exercise. The last minute consisted of the last four data points, not including the final data point, of the 15-second moving average smoothed data. The median, maximum, and minimum energy expenditure values for each fundamental movement computed over 1 minute are shown in Table 2.

**Table 2:** Steady-State Energy Expenditure over 1-minute period.

Activity (*n* = 19)	Median	Max.	Min.
Standing Jump (kJ)	41.7925	59.2362	10.3225
Arm Swing (kJ)	9.8870	25.9959	4.1720
Squat (kJ)	15.1821	24.1215	8.4139
Jumping Jack (kJ)	47.3117	80.5382	34.4308

### Mechanical work results

**Fig 5:**
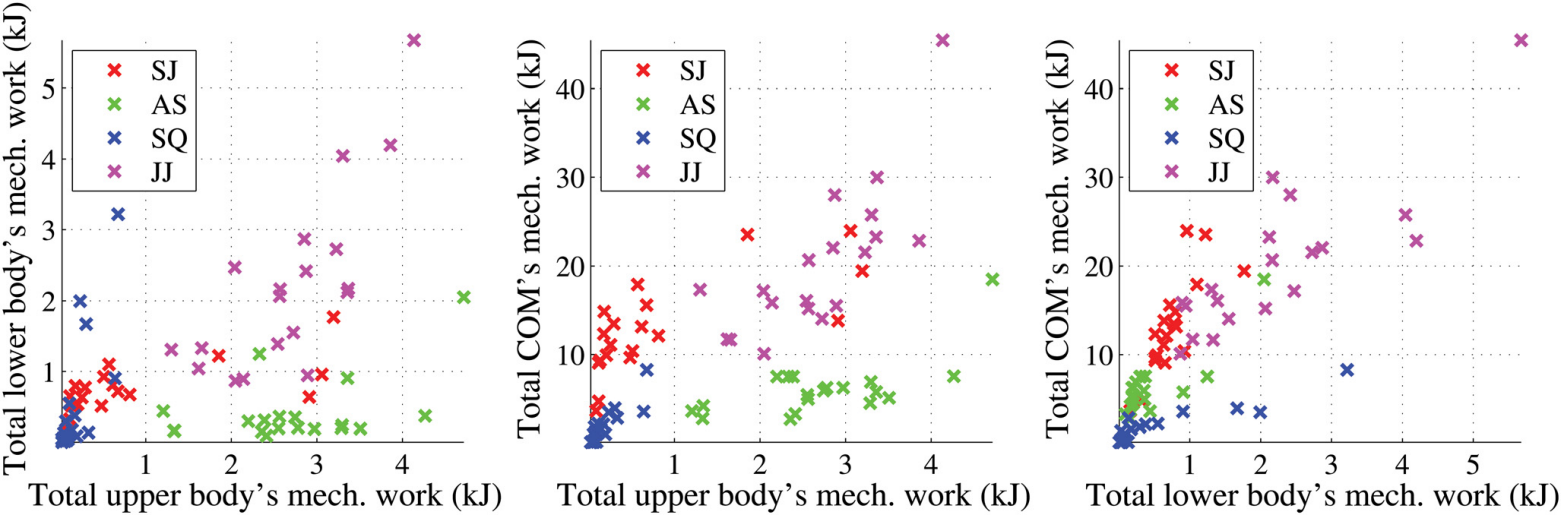
Scatter plots showing relationships among the total mechanical works of upper body, lower body, and centre of mass.

Table 3 shows the mean mechanical work calculations for the four fundamental movements that were used to predict energy expenditure. The first 6 columns in the table denote the mean of the 6D feature vectors. These columns and the last column, which corresponds to the posture cost, in the table denote the mean of the 7D feature vectors, while the *Sum* column alone denotes the mean of the 1D feature vectors used in our experiments. The Excel spreadsheet containing the components of the 7D feature vectors of all the participants and their corresponding metabolic energy values is given in S1 Data. Fig 5 shows the scatter plots of the total mechanical work of the upper body, lower body, and centre of mass for the 76 subjects. The total upper body’s mechanical work is computed to be the sum of the positive and negative upper body’s mechanical works (no cancellation as both are positive values). The total lower body’s and centre of mass’ mechanical works are computed in a similar fashion. Each of the four fundamental movements represents a different activity mode with the distribution of mechanical work among the upper body, lower body, centre of mass, and posture. In particular, we can see the high *upper body* and low *centre of mass* work of the *Arm Swing*, the high *posture cost* of the *Squat* and the high *centre of mass* work for both *Standing Jump* and *Jumping Jack*.

**Table 3:** The mean mechanical work with posture cost for the four fundamental movements over 1-minute period.

Activity (*n* = 19)	Upper body		Lower body		Centre of mass		Sum	Posture
	Pos	Neg	Pos	Neg	Pos	Neg		
Standing Jump (kJ)	0.4376	0.4157	0.3735	0.3756	6.5123	6.5368	**14.6515**	0.2579
Arm Swing (kJ)	1.3573	1.3575	0.2137	0.2145	3.0402	3.0746	**9.2578**	0.0091
Squat (kJ)	0.0872	0.0871	0.2591	0.2608	0.9547	0.8636	**2.5125**	2.9437
Jumping Jack (kJ)	1.3414	1.3459	1.1117	1.1115	10.0798	10.1351	**25.1253**	0

### Predictive model results

**Fig 6:**
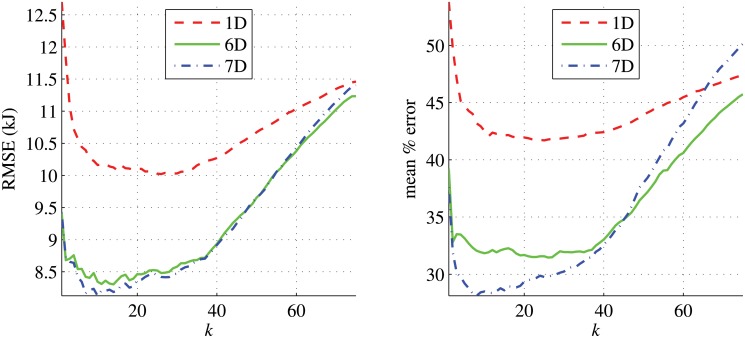
The RMSE and mean percentage errors of the predicted energy costs from KNNR for different values of *k*.

We evaluated the three regression algorithms, GPR, KNNR, and LINR, on the 1D, 6D, and 7D feature vectors. To obtain the most suitable value of *k* for KNNR on our data, we experimentally evaluated the performance of the regression algorithm using the weight function $w(\tilde{\text{x}},{\text{x}}_{*})=1/d(\tilde{\text{x}},{\text{x}}_{*})$ for different values of *k*. Fig 6 shows that, for all the 1D, 6D, and 7D feature vectors, the smallest value of *k* that gives the lowest RMSE and mean percentage error is around 10 for the 7D feature vectors. For the 1D and 6D feature vectors, any *k* value in the interval [10, 30] appears to give similar mean percentage errors. We thus chose *k* = 10 in all our experiments.

**Fig 7:**
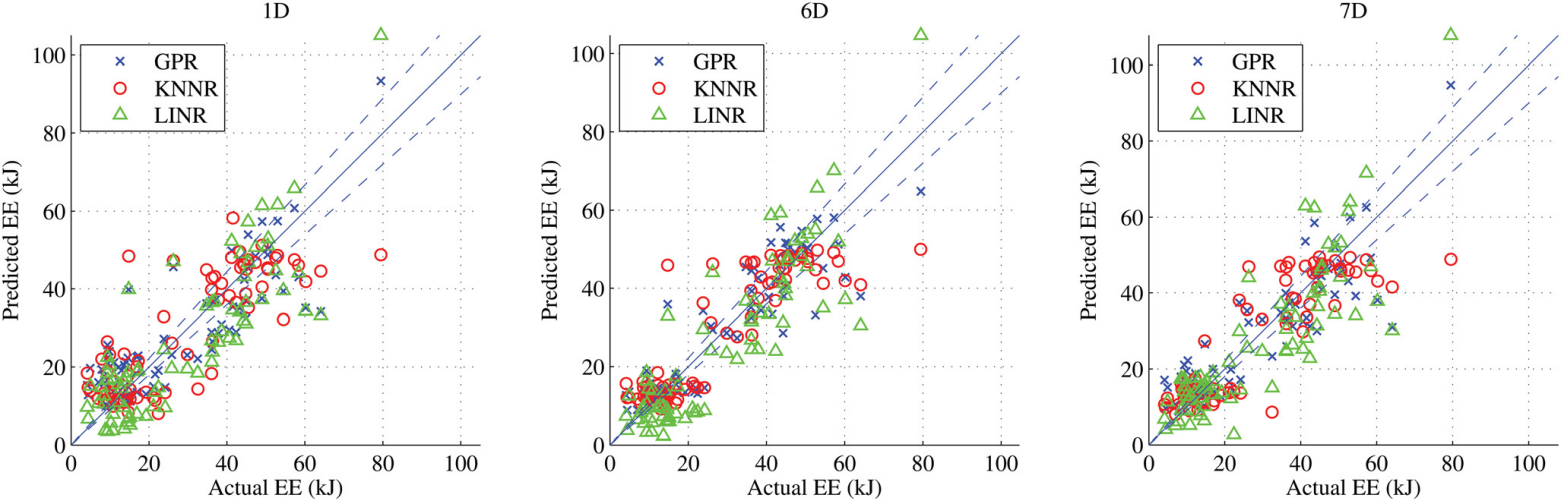
The predicted energies from the three algorithms versus the actual energies for the 3 feature types 1D, 6D, and 7D. The blue diagonal line denotes the 45° line. The two diagonal blue dashed lines denote the ±10% error about the 45° line.

**Fig 8:**
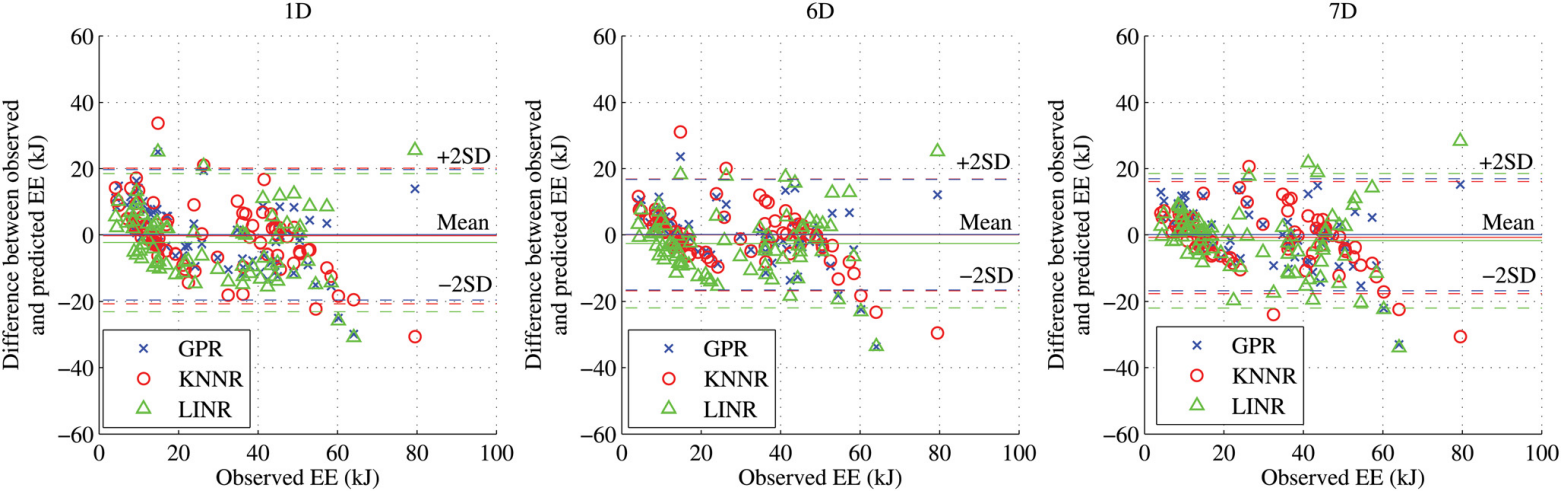
The Bland-Altman plots for 3 features types. The differences between observed and predicted values from the three algorithms versus the actual observed energies for the 3 feature types 1D, 6D, and 7D. The solid line is the mean of the differences for the 3 features types, while the dotted lines denote ±2 standard deviations from the mean.

RMSE of predictions from GPR, KNNR, and LINR for the three feature types are shown in Fig 7. Using the LOOCV technique, we compared the predicted metabolic energy costs with the ground truth values. We also present the Bland-Altman diagnostic plots for the three feature types in Fig 8. The Bland-Altman diagnostic plots are used to assess the agreement between measurement methods [42]. This is done by displaying the difference between the two measurements along with the mean and two standard deviation bounds. The performances of these algorithms are very similar. There is good agreement for all feature types in the Bland-Altman diagnostic plots in Fig 8. The RMSE, percentage errors, and concordance correlation coefficients (CCC) of the predictions from these algorithms are shown in Table 4. For the 1D features, GPR gave the smallest RMSE but LINR gave the smallest percentage error and highest CCC. For the 6D and 7D features, GPR gave the smallest RMSE and largest CCC, but LINR gave the smallest mean percentage error of prediction for the 7D features.

**Table 4:** The RMSE (in kJ), mean percentage errors, and concordance correlation coefficients (CCC) of the GPR, KNNR, and LINR algorithms for the three feature types. The figures in bold denote the smallest RMSE, percentage errors, or the largest concordance correlation coefficients.

Algorithm	1D			6D			7D		
	RMSE	%error	CCC	RMSE	% error	CCC	RMSE	% error	CCC
GPR	**9.759**	46.00%	0.827	**8.276**	32.61%	**0.882**	**8.384**	35.64%	**0.879**
KNNR	10.163	42.53%	0.813	8.344	**31.84%**	0.876	8.415	29.76%	0.847
LINR	10.596	**41.63%**	**0.833**	9.975	33.65%	0.864	10.229	**29.39%**	0.847

To assess whether the differences in performances of the three algorithms are significant statistically, we paired up the three algorithms and performed the paired t-tests on their predicted metabolic energy costs. For each t-test, we used the null hypothesis ℋ_0_ which asserts that the two sets of metabolic energy costs came from the same distribution. The results are shown in Table 5. When GPR was compared against KNNR, ℋ_0_ was not rejected for all the feature types, indicating no difference between the performances of GPR and KNNR. However, when GPR was compared against LINR, ℋ_0_ was rejected for all the feature types. The results in Table 5 show that, apart from the 6D features, the performances of LINR and KNNR had no difference statistically.

**Table 5:** The paired t-test results on the three algorithms with the Null hypothesis ℋ_0_: the predicted energy values from the two algorithms being considered come from the same distribution.

Algorithms	1D		6D		7D	
	Result	*p*-value	Result	*p*-value	Result	*p*-value
GPR vs KNNR	Failed to reject ℋ_0_	0.72	Failed to reject ℋ_0_	0.94	Failed to reject ℋ_0_	0.34
GPR vs LINR	Rejected ℋ_0_	7.7 × 10^−8^	Rejected ℋ_0_	2.4 × 10^−5^	Rejected ℋ_0_	0.01
KNNR vs LINR	Failed to reject ℋ_0_	0.08	Rejected ℋ_0_	1.9 × 10^−2^	Failed to reject ℋ_0_	0.42

**Fig 9:**
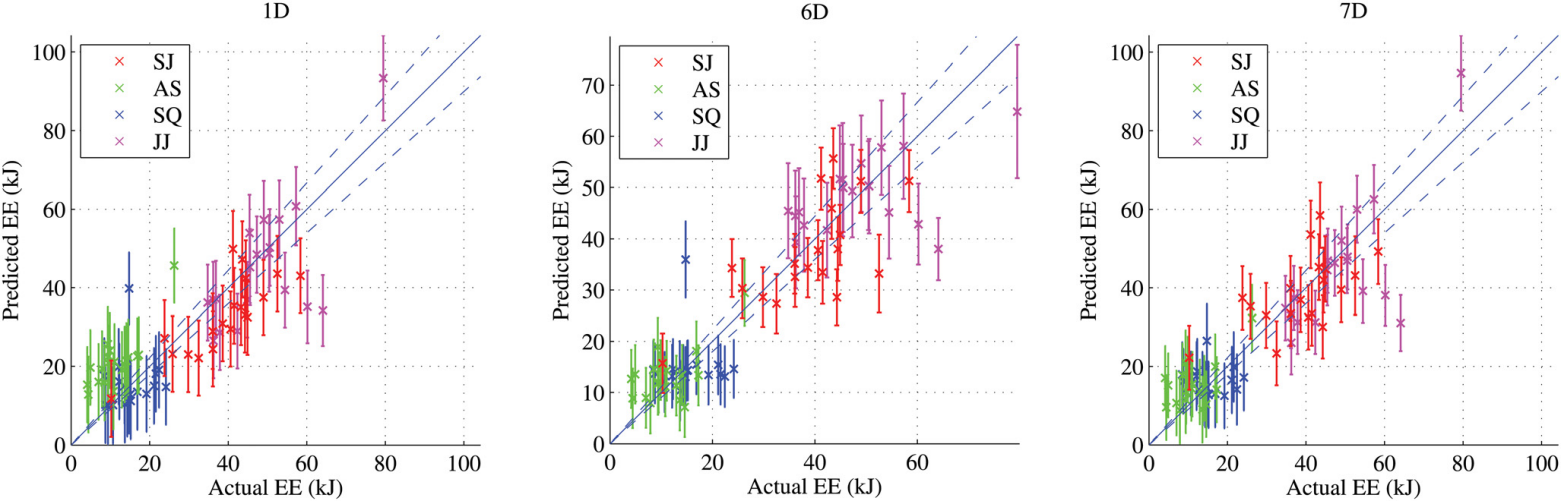
The predicted energies from GPR versus the actual energies for the individual fundamental movements. The vertical error bars denote one standard deviation on either side of the mean. The results were obtained by using the LOOCV technique on all the movements together.

Unlike KNNR and LINR, the GPR also produced the variance of each prediction. As shown in Fig 9, the standard deviations were around 8 kJ. When the actual energy is low, this 8 kJ standard deviation corresponds to a large uncertainty in the predicted value. We found that the performance of GPR could be slightly improved if the *Jumping Jack* movement was trained and tested separately from the other three movements, e.g., for the 6D and 7D features, the RMSE and mean percentage error dropped to 7.54 kJ and 27.7% respectively. This improvement in prediction was expected as the actual energy values of the *Jumping Jack* movement are much larger than those of other movements. With only 76 samples for each feature type, training and predicting the *Jumping Jack* movement separately allows the algorithm to focus locally on those regions of high energy values.

For KNNR, we found that using the weighting function $w(\tilde{\text{x}},{\text{x}}_{*})=1/d^{2}(\tilde{\text{x}},{\text{x}}_{*})$ caused the performance to drop. With *k* again set to 10 for the 1D features, the RMSE of the predicted metabolic costs increased to 11.24 kJ and the mean percentage error increased to 45.6% when this new weighting function was used. A similar reduction in performance was observed for the 6D and 7D features. Using the weighting function $w(\tilde{\text{x}},{\text{x}}_{*})=\exp(-d^{2}(\tilde{\text{x}},{\text{x}}_{*})/b)$, with *b* = 1000, the KNNR gave slightly smaller RMSE and mean percentage errors for all the three feature types. However, the improvements were not statistically significant.

The RMSE shown in Table 4 were obtained from the data of all the four activities put together. If these errors were separated based on the activity type (Table 6), then LINR gave the lowest RMSE for all feature types for Arm Swings but performed poorly on other activities. Out of the three algorithms, the RMSE of GPR (and KNNR) were the lowest in 5 (and 4) out of 12 cases (combination of 4 activities over 3 feature types). This is a 42% (and 33%) share for the twelve cases. When GPR took the second place, the RMSE it produced is very close to the smallest RMSE for the majority of the cases. In general, there are improvements in the predictions for all algorithms for the Standing Jumps, Arm Swings, and Squats when moving from the univariate 1D model to the multivariate 6D model. For the Jumping Jacks, the 1D, 6D and 7D feature vectors all have similar performance. Moreover, the addition of a posture cost in the 7D feature vector does improve the predictive performance for static poses, such as squatting, but appears to affect the predictive performance of the Standing Jumps. This demonstrates that, if the activity type is known, energy expenditure predictions can be improved for static poses with high joint torques by appropriately defining the posture cost.

**Table 6:** The RMSE (in kJ) for the four activities standing jump (SJ), arm swing (AS), squat (SQ) and jumping jack (JJ).

Activity	1D			2D			3D		
	GPR	KNNR	LINR	GPR	KNNR	LINR	GPR	KNNR	LINR
SJ	8.792	8.685	10.179	6.972	5.429	8.065	8.927	9.064	11.367
AS	10.746	9.519	7.743	6.136	6.852	5.785	6.385	6.213	5.811
SQ	7.370	9.987	9.926	6.856	8.472	10.011	5.678	6.037	6.779
JJ	11.571	12.139	13.676	11.860	11.415	14.116	11.335	11.228	14.479

## Discussion

We have demonstrated how physical activity energy expenditure during exercise, using the Kinect camera as a motion capture system, can be estimated from segmental mechanical work. This was achieved by employing machine learning and regression techniques, such as GPR, KNNR, and LINR, on the mechanical work distributed to the different body segments in a variety of exercises. As exercises chosen in our study varied greatly in intensity, our experiments covered the estimation of a large range of energy expenditures. Our research finding suggest that the Kinect sensor could be used to estimate energy expenditure during active video gaming, as well as provide feedback within the gaming environment to influence game play and energy expenditure at the body segment level.

To accurately attribute physiological cost to a specific mechanical movement, we designed experiments so that individual exercises were repeated to reach a steady-state of oxygen uptake. The timing of each exercise was regulated by using a metronome. This was necessary because it is only at steady-state exercise that the balance between the energy required by the muscles and the energy that is produced by aerobic metabolism is represented by $\dot{\text{V}}{\text{O}}_{2}$ [43]. The difficulty of controlling exercise without an external load or incremental speed change to reach steady-state meant that $\dot{\text{V}}{\text{O}}_{2}$ varied within each activity. Nevertheless, there is a distinct difference in the $\dot{\text{V}}{\text{O}}_{2}$ profile for each activity as demonstrated in Fig 4. The limited energy expenditure that comes from upper body movements is demonstrated in the $\dot{\text{V}}{\text{O}}_{2}$ profile of Arm Swings compared to the other activities. The use of 5-second breaks within the Standing Jump also has a notable effect on its $\dot{\text{V}}{\text{O}}_{2}$ profile. There is little work on best practices for $\dot{\text{V}}{\text{O}}_{2}$ measurements for these activity modes and so further investigation into experiment protocol and design is needed.

We have also presented a novel application of the centre of mass (COM) model [28] of mechanical work. While the COM model is usually restricted to locomotion activities, such as walking or running, we apply the model to other activity modes. Our four fundamental movement types were *Standing Jumps*, *Arm Swings*, body-weight *Squats* (or knee bends) and *Jumping Jacks*. Since some of these activities involved static poses that require joint torques which would not present any mechanical work, a posture cost based on the ankle-to-hip distance relative to the leg length was implemented. The inclusion of the posture cost for squatting (7D in Table 4) shows how static pose indicators can be used to improve energy expenditure predictions. External work measurements in the COM model are usually made with force platforms, but kinematic measurements alone have been shown to have negligible effect on the precision of the measurements [18]. External work calculations are dependent on a global coordinate system, which in the case of walking or running, is usually taken to be the projection of the COM position onto the ground plane. In our case, since the activities were not locomotion-focused (i.e., the participant was not required to move to another position relative to the camera), this would have had a limited impact on the variation in external work values.

Rather than calculating a total mechanical work value, we implemented a multivariate model, aggregating mechanical work according to the positive and negative work and limb type. While the distribution of mechanical work across limbs is consistent during locomotion activities, in other activity modes the locus of mechanical work contains important information about the relative metabolic cost of the exerted total mechanical energy. For example, in many exercises the arms will have a lower metabolic cost than the legs. The relative cost in these segments can be predicted from an independent estimate of the mechanical work in each of these segments. This information of the mechanical work performed by different limbs is lost when summed to form a total mechanical work measurement. This is why upper body arm movements are usually left out or combined to form a Head, Arms, Trunk (HAT) segment in total mechanical work calculations for walking or running [28, 32]. How these mechanical work values interact to impact metabolic energy is also especially complex as it is only for very specific movements that it is possible to precisely attribute a mechanical work value to muscular work [27]. Thus, treating the problem of modelling the dependency of metabolic cost on mechanical work as a multivariate problem that can be captured using a complex flexible nonparametric model is conceivably appropriate.

We have demonstrated how nonparametric techniques, such as GPR and KNNR, can improve the predictive ability of a linear model. Statistical learning and algorithmic data modelling are approaches to function estimation that sets certain conditions on how generalizable a model is to new data [19, 44]. The statistical learning approach of fitting a model to a set of data, rather than assessing the fit of a dataset to a model, requires that there be some way of quantifying how good is a model’s generalization performance [38]. This leads to the concept of cross-validation in assessing the generalizability of a statistical model. In our case, we implemented a leave-one out cross-validation (LOOCV) so that the generalizability of the model to each data point was tested. The root-mean squared error (RMSE) and relative mean percentage error of the LOOCV was used to compare different techniques’ performance. The smaller RMSE values for the 6D and 7D features in Table 4 demonstrated that the predictive ability of the nonparametric GPR and KNNR models consistently outperformed the linear regression model.

With advances in active video gaming technology, such as the Microsoft Kinect Sensor, the potential to advance the measurement and requirements of energy expenditure during gameplay have been greatly enhanced. The results of this study offer the first attempt to demonstrate how mechanical work measurements using the Microsoft Kinect depth camera and skeletal tracking algorithm can be used to make estimations of energy expenditure. With active video game health benefits linked to both duration and exercise intensity, such accurate measurements are important. In this study we focused on demonstrating our approach on the steady state condition in the context of active video gaming. As our results do a reasonably good job in predicting energy use in steady state conditions, future research could use this as an approach to assess energy use of discrete and short duration movements from the mechanical data from the Kinect Sensor. It should be noted that the Microsoft Kinect-v2 is capable of monitoring heart rate and can thus provide supplementary information about cardiovascular activity. It is possible that the new Kinect will provide greater sensitivity to skeletal measures and possible additional physiological data that could further refine the model. Furthermore, the approach in the current study is a big step forward for measuring steady state activity during active video game play. Future research could focus on extending the current research to explore the potential for assessing activities during periods of low activity in addition to steady state exercise.

We have shown that for high-energy activities, such as *Standing Jumps* or *Jumping Jacks*, estimates can be made accurately, but that for low-energy activities such as *Squatting* the posture of static poses should be considered as a contributing factor. Moreover, we have demonstrated how a multivariate statistical learning approach can predict energy expenditure from mechanical work. These predictions are possible without any prior information except for the subject’s body mass and height. When translated into the active video gaming environment, the results of this study represent an opportunity to inform designers of how energy is expended in various movement tasks during active video game play.

## Supporting Information

S1 Data Excel spreadsheet containing the 7D feature vectors and metabolic energies of the participants. (XLSX)
